# Respiratory Effects of Simultaneous Exposure to Respirable Crystalline Silica Dust, Formaldehyde, and Triethylamine of a Group of Foundry Workers

**Published:** 2017-03-04

**Authors:** Fatemeh Zarei, Mansour Rezazadeh Azari, Sousan Salehpour, Soheila Khodakarim, Leila Omidi, Elahe Tavakol

**Affiliations:** ^1^ Department of Occupational Health Engineering, School of Public Health, Shahid Beheshti University of Medical Science, Tehran, Iran; ^2^ Safety Promotion and Injury Prevention Research Center and Department of Occupational Health Engineering, School of Public Health, Shahid Beheshti University of Medical Science, Tehran, Iran; ^3^ Department of Epidemiology, School of Public Health, Shahid Beheshti University of Medical of Medical Sciences, Tehran, Iran; ^4^ Department of Biostatistics, School of Paramedical Sciences, Shahid Beheshti University of Medical of Medical Sciences, Tehran, Iran; ^5^ Department of Occupational Health Engineering, School of Public Health, Tehran University of Medical Sciences, Tehran, Iran

**Keywords:** Workers, Lung Function, Exposure, Occupational

## Abstract

**Background:** Foundry workers are occupationally exposed to hazardous substances such as silica dusts and toxic gases. The aim of this study was to examine the effects of simultaneous exposure to complex mixtures of silica dust, formaldehyde, and triethylamine on lung function parameters.

**Study design:** A cross-sectional study.

**Methods:** This study was conducted on 55 male workers of core making unit of a foundry plant (the case group) and 55 workers in a food industry were enrolled as a control group in 2015. Workers were monitored for personal exposure to crystalline silica respirable dust, according the NIOSH method No.7602. The concentrations of formaldehyde and triethylamine were measured using a PID instrument. Lung function tests were performed according to the ERS/ATS standards.

**Results:** The mean concentrations of personal exposure to silica dust, formaldehyde, and triethylamine in the core making workers were 0.23 mg/m^3^ , 2.85 ppm, and 5.55 ppm and respective exposures of control subjects were less than the LOD (limit of detection). There were significant associations between exposure to silica dust and decreases in FVC (Forced vital capacity) values (*P*<0.05). The findings showed a statistically significant synergistic effect of silica dust and triethylamine on FVC values (*P*<0.05).

**Conclusions:** The mean exposure of all studied substances was higher than occupational exposure limits. Synergistic effects of exposure to silica dust and triethylamine on some lung function parameters were observed. Simultaneous exposure of foundry workers to silica dust and triethylamine could impair lung function.

## Introduction

Workers in many workplace settings are occupationally exposed to a number of hazardous substances, associated with a wide range of adverse health effects^1, 2^. Exposure to silica dust in foundry workers has been described previously^3^. Exposure to crystalline silica has caused silicosis, lung cancer, pulmonary tuberculosis, chronic bronchitis, and chronic obstructive pulmonary disease^4-6^. International Agency for Research on Cancer (IARC) classified inhaled crystalline silica as a group 1 carcinogen^7, 8^.

Westberg and Bellander^9^conducted a case**-**control study in a cohort of 5016 foundry workers employed for at least one year in the studied aluminum foundries in Sweden including 31 cases of lung cancer. Their results indicate the odds ratio of lung cancer of cumulative quartz exposures in the range of 0.001–1.0 mg/m^3^**-**yr at a value of 1.6 (95% confidence interval of 0.54 to 4.6). The geometric mean value for occupational exposure to crystalline silica aerosols in 20 studied foundry workers in the east zone of Tehran was 0.343 mg per cubic meter^10^. The concentration of respirable crystalline silica dust in aluminum foundry workers was 0.10 mg per cubic meter^11^. The air concentrations of respirable silica dust in the studied stone crushing units in Iran exceeded the threshold limit value (TLV) in industrial units of Azandarian^12^.

Several attempts have been made to measure pulmonary function among foundry workers. Koo et al. ^13^ studied the effect of exposure to silica dust on lung function tests of 209 male foundry workers and 239 office workers selected as a control group. Geometric mean concentration of the quartz in molding and core making units were 0.051 mg/m^3^ and 0.023 mg/m^3^, respectively. All ventilation parameters except FVC were significantly lower in foundry workers than those in the control group were. The reduction in the FVC and FEV1 values of workers exposed to respirable crystalline silica dust was reported^14^.

Formaldehyde as a highly reactive carbonyl compound may cause irritation and health symptoms^15^. Exposed workers were reported to suffer from irritation of the upper respiratory system, change in pulmonary function parameters such as decline in the FEV1, and sensitization effects^16, 17^. IARC classified formaldehyde as human carcinogens that cause nasopharyngeal cancer in humans^18, 19^. Reversible changes in the lung function parameters have been observed at 2.4 mg/m^3^ exposure level^20^.

Tertiary amines such as triethylamine are used in the core making processes^21^. The general effects of tertiary amines include irritation, headache, nausea, anxiety, faintness and corrosive effects to the skin, mucous membrane, eye, nose, throat, respiratory distress, cough, and corneal edema^22^.

Given the importance of adverse health effects of workers' exposure, there has been little discussion on the effects of simultaneous exposure to complex mixtures of silica dust, formaldehyde, and triethylamine on the respiratory system of foundry workers abroad and in Iran.

Therefore, the objective of this study was to examine the effects of simultaneous exposure to complex mixtures of silica dust, formaldehyde, and triethylamine on the respiratory system of core making workers at a foundry located in the greater Tehran, Iran.

## Methods

### Study participants

Census procedure was used to determine the sample size and all 55 male workers of core making unit of a foundry plant (occupationally exposed to a mixture of silica dust, formaldehyde, and triethylamine), located in Tehran, Iran with inclusion criteria of one year of work experience and willing to participate in the research were enrolled in this cross-sectional analytical study in 2015 (the case group). Fifty**-**five male subjects of a food manufacturing plant without active exposure to any dusts or gases from similar socioeconomic class participated as a control group as well. Workers and controls were matched for gender and the number of participants. The demographic features such as age, work experience, body mass index (BMI) of workers, and cigarette smoking habit in the case and control groups were obtained prior to survey. None of the exposed workers used personal protective equipment.

All subjects signed consent forms and the study was approved by local Ethics Committee.

### Sampling procedure

Since, the concentration of air pollutants is affected by indoor climate parameters, indoor climate factors, including relative humidity, air pressure level, and wind speed were measured in core making environment.

Workers' exposure to crystalline silica dust was monitored according to an improved method of National Institute for Occupational Safety and Health (NIOSH) 7602^23, 24^. Each set was comprised of a calibrated SKC sampling pump (model Deluxe, SKC, UK) equipped with mixed cellulose ester (MCE) filters (SKC Inc., USA) and a 10-mm nylon cyclone (SKC) at a flow rate of 1.7 L/min. Quartz purchased from Merck (Germany) was used for the preparation of standard samples in the range of 20-200 µg per sample. After personal sampling, 200 mg potassium bromide purchased from Merck Co. was added to MCE filters. All filters were placed in the muffle furnace at 600 °C for 2 h. Subsequently, all samples were transferred to a mortar for complete mixing and homogenizing. Finally, each sample was pressed under 20 mega-pascal for 2 min for manufacturing 13 mm pellets. Each pellet was analyzed for crystalline silica by WQF-510A FT-IR spectrometer (China) in a range from 400 to 4000 cm^-1^ or 710-825 nm^24^.

The validity of silica dust measurements was determined as well^25^. The intra- and inter-day coefficients of variation were considered for the precision of the method and accuracy was examined as percentage of recovery of standards. The coefficient of variation less than 10% and recovery efficiency in the range of 80% to 100% was considered as acceptable^26^. The LOD and LOQ (limit of quantification) values for analysis of crystalline silica dust were calculated according to equation No. 1 and No. 2, respectively.

*LOD = Xb*_i_ *+ 3Sb*_i_ Eq. 1

*LOQ = Xb*_i_ *+ 10Sb*_i_ Eq. 2

Where Xb_i_ was the mean concentration of the blank and Sbi was the standard deviation (SD) of the blank^27^.

The concentrations of formaldehyde and triethylamine were measured using a photoionization detector (PID) apparatus (PhoCheck Tiger, Ion Science Inc., UK). The usual mechanism of action of PID instrument as a real-time analyzer is ionization using ultraviolet radiation^28^. PhoCheck Tiger PID was used to measure some compounds in previous work such as the toluene concentration at the inlet and outlet of the oxidation reactor^29^. The instrument was calibrated using the contamination-free air in the range of 0.4 to 14 ppm prepared in Tedlar bags (SKC Inc., USA). Atmospheric stock gas standards were prepared through static injection of known amounts of volatile liquid (using micro syringe Hamilton Company) into a 1L Tedlar bag containing contamination-free air. Hamilton gas tight syringe (0-500 µl) was used to transfer known amount of gas volume into 10 L Tedlar bags and filling them with known volume of contamination-free air by setting the proper flow and time parameters. Contamination-free atmosphere gases in the range of 0.4 to 14 and 0.4 to 12 as ppm for formaldehyde and trimethylamine respectively were prepared for calibration of PID.

Lung function tests were performed using a spirometer (Cardio Touch 3000, USA). Pulmonary function parameters such as FVC, FEV1, FEV1/FVC, peak expiratory flow (PEF), and mid forced expiratory flow (FEF 25–75%) were considered. Lung function tests were measured according to the ERS/ATS standards before shift (during 6**-**7 a.m.) and after shift (during 4**-**5 p.m.). The workers' state of respiratory health was classified according to ATS/ERS guidelines for spirometric patterns of normal, obstructed, restricted, or mixed obstructed and restricted^30-32^. The subjects rested for 5 min before performing pulmonary**-**function tests. Spirometry was performed in standing positions of subjects and at least three acceptable maneuvers were obtained. Spirometric values were corrected for age, height, weight, and smoking habit^31.^

### Statistical analysis

All statistical tests were performed with SPSS 18 for Windows (SPSS Inc., IL, USA). The normality of the data was determined using Kolmogorov–Smirnov test. The independent *t***-**test was used to determine the difference between the quantitative variables. Significant differences between the number of cigarettes smoked per day by workers in the case and control groups were determined using the Mann**-**Whitney U test. The association between the exposures to studied compounds and lung function parameters were examined by regression analysis. The *P*-value at <0.05 was considered significant.

## Results

The average (SD) regarding age, work experience, and BMI of core making workers was 31.98 (6.90) yr, 5.32 (4.59) yr, and 25.11 (3.82) kg/m^2^, respectively. Among core making workers, 32.7% smoked cigarettes. The average (SD) number of cigarettes smoked per day was 11.56 (7) during 10.81 (7.33) yr on average. The average (SD) regarding age, work experience, and BMI of workers in the control group were 37.15 (7.96) yr, 10.72 (6.84) yr, and 25.24 (3.77) kg/m^2^, respectively. Among subjects in the control group, 14.5% smoked cigarettes. The average (SD) number of cigarettes smoked per day was 7.50 (5.34) during 8.38 (5.29) yr on average. The independent samples *t***-**test indicated significant differences in age and work experience of workers in the case and control groups (*P*<0.001). The chi**-**square test revealed that the numbers of current smokers were significantly higher (*P*=0.025) in the case group than in the control group. Because the spirometric values were based on subjects' age in the case and control groups, no adjustment was applied to this variable. The results were adjusted for work experience and the number of cigarette smokers. However, no statistical significant differences were found for other demographic variables.

Measured indoor air parameters in the core-making unit of a foundry plant are presented in Table 1. The mean relative humidity in the studied unit was 58.15%. The intra and inter-day coefficients of variation for measuring silica dust using NIOSH 7602 FT**-**IR spectroscopy method was 2.57 and 3.61%, respectively. The efficiency of recovery for silica dust samples was 100%. The LOD and LOQ for silica dust samples were 5.04 μg/sample and 3.82μg/sample, respectively.

**Table 1:** Indoor air parameters in the core-making unit

**Indoor air parameters**	**Mean**	**SD**	**Range**
Air temperature (°C)	26.51	3.70	20 to 33
Air velocity (m/s)	0.58	0.12	0.34 to 0.79
Air pressure (mmHg)	649.33	1.25	647 to 651

The results obtained from the monitoring of occupational exposure to crystalline silica, formaldehyde, and triethylamine in the core-making unit are shown in Table 2. The findings were compared with respective threshold limit values proposed by ACGIH and the Iranian occupational exposure limits (OELs)^33, 34^.

**Table 2:** Exposure information for workers of core making unit

**Personal exposure data**	**Mean**	**SE**	**Range**	**Threshold** **limit values**
Silica dust (mg/m^3^)	0.25	0.05	0.05 to 2.40	0.025
Formaldehyde (ppm)	2.91	0.18	0.41 to 6.47	0.300
Triethylamine (ppm)	5.24	0.22	2.83 to 8.06	1.000

Results of lung function tests for exposed and control groups were compared (Table 3). However, statistically significant differences were found for demographic parameters of exposed and control group such as work experience and the number of smokers. For comparing the lung function parameters in the exposed and control groups, the results were adjusted for work experience and the number of cigarette smokers. The paired *t***-**test showed significant decreases (*P* <0.05) in the mean values of some lung function parameters including FVC, FEV1, and FEV1/FVC values in the exposed group compared to control group for before and after work shifts. Linear regression (for comparing the mean values of lung function parameters) indicated significant differences (*P*<0.05) in FVC and FEV1 values between exposed and control subjects before and after work shifts.

**Table 3:** Comparison of the lung function test results in the case and control groups

**Parameters**	**Exposed group**			**Control group**			***P *** **value**
	**Mean (±SD)**	**Range**	***P*** ** value**	**Mean (±SD)**	**Range**	***P*** ** value**	
FVC (%)			0.001			0.001	
Before	77.45 (12.43)	43.20 to109.29		84.35 (10.31)	64.80 to 131.10		0.005
After	72.18 (13.10)	34.80 to 101.25		82.24 (10.46)	63.92 to 121.26		0.001
FEV1 (%)			0.001			0.333	
Before	78.10 (10.46)	52.80 to 111.46		83.98 (9.70)	58.40 to 127.7		0.017
After	74.06 (11.77)	41.30 to 110.24		83.38 (9.98)	67.14 to 127		0.001
FEV1/FVC (%)			0.001			0.001	
Before	84.59 (8.71)	60.52 to 99.99		82.84 (6.94)	67.50 to 99.99		0.246
After	86.21 (9.81)	60.22 to 99.99		84.53 (6.84)	67.50 to 99.99		0.654
FEF 25–75%			0.769			0.141	
Before	86.59 (30.55)	24.88 to 186.60		94.47 (34.42)	41.90 to 256.90		0.638
After	85.85 (32.66)	24.88 to 195.80		99.40 (34.26)	35.30 to 256.70		0.167
PEF (%)			0.001			0.409	
Before	84.20 (12.42)	61.40 to121.87		88.27 (19.62)	51.60 to 183.30		0.179
After	80.54 (13.20)	51.60 to 110.30		86.52 (15.04)	58.71 to 129.20		0.165

Note: FVC: forced vital capacity; FEV1: forced expiratory volume for one second; FEF 25-75%: expiratory flow between 25% and 75% of vital capacity; PEF: peak expiratory flow

Mann**-**Whitney tests revealed that differences in the spirometric patterns of workers between the exposed and control groups before starting a work shift (*P*=0.001) and after the work shift (*P*=0.007) were significant (Table 4).

**Table 4:** Comparison of the spirometric patterns in the case and control groups

**Work shift**	**Exposed group, n (%)**				**Control group, n (%)**				***P*** ** value**
	**Normal**	**Obstructive**	**Restrictive**	**Mixed**	**Normal**	**Obstructive**	**Restrictive**	**Mixed**	
Before	22 (40.0)	5 (9.1)	13 (23.6)	15 (27.3)	41 (74.5)	4 (7.3)	8 (14.5)	2 (3.6)	0.001
After	14 (25.5)	5 (9.1)	18 (32.7)	18 (32.7)	40 (72.7)	4 (7.3)	9 (16.4)	2 (3.6)	0.007
*P* value	0.046				0.154				

The association of lung function parameters with the amount of exposures to silica dust, formaldehyde, and triethylamine was checked by regression analysis. A negative sign of the partial regression coefficient (β) denotes that increases for exposure to silica dust and triethylamine were associated with decreases in FEV1 and FVC values (Table 5). No significant associations were observed for formaldehyde.

**Table 5:** The association of lung function parameters with exposures data in the case group

**Contaminants**	**Before**						**After**					
	**FVC (%)**			**FEV1 (%)**			**FVC (%)**			**FEV1 (%)**		
	**β**	**SE**	***P*** ** value**	**β**	**SE**	***P*** ** value**	**β**	**SE**	**P**	**β**	**SE**	***P*** ** value**
Silica dust	**-**0.355	0.004	0.008	**-**0.269	0.004	0.047	**-**0.387	0.003	0.004	**-**0.349	0.004	0.009
Formaldehyde	**-**0.138	1.245	0.313	**-**0.460	1.057	0.737	**-**0.205	1.297	0.133	**-**0.136	1.179	0.322
Triethylamine	**-**0.258	0.976	0.057	**-**0.158	0.840	0.250	**-**0.286	0.017	0.034	**-**0.258	0.924	0.057

Note: FVC: forced vital capacity; FEV1: forced expiratory volume for one second; β: partial regression coefficient; SE: standard error.

The association of lung function parameters with exposures to silica dust was determined after adjusting for exposures to formaldehyde, and triethylamine. The multiple regression analysis of data revealed that there were significant associations between exposure to silica dust and FVC values before shift (*P*=0.034; β=**-**0.301) and after shift (*P*=0.023; β=**-**0.317). There were significant associations between exposure to silica dust and FEV_1_ values only after shift (*P*=0.038; β=**-**0.295).

he synergistic effects of simultaneous exposures to silica dust, formaldehyde, and triethylamine were investigated using linear regression analysis. The findings showed a statistically significant synergistic effect of silica dust and triethylamine on FVC values before (*P*=0.025) and after (*P*=0.029) work shifts. In addition, the results indicated a statistically significant synergistic effect of silica dust and triethylamine on FEV1 (*P*=0.010) and PEF (*P*=0.009) before and after work shifts FEV_1_ (*P*=0.001); PEF (*P*=0.003). The statistically significant synergistic effect of exposures to formaldehyde and smoking habits on FEF 25–75% values before (*P*=0.036) and after (*P*=0.028) work shifts were noticed.

The simple regression analysis of cumulative exposure (exposure*years of work) data indicated that there were significant associations between exposure to silica dust and FVC, FEV1, and FEF 25–75% values before and after work shifts (*P*<0.05).

## Discussion

Data from this study indicated that all 55-foundry workers were exposed to respirable silica dust, formaldehyde, and triethylamine having a higher exposure than respective TLV proposed by ACGIH^33^and Iranian OEL^34^. The average concentration of respirable silica dust for foundry workers in the current study was less than Swedish foundry workers during the periods 1970–1979, but, higher than same groups of workers during the 1980–1989 periods^9^.

The occupational exposure of foundry workers in this study (0.41-6.47 ppm) to formaldehyde was approximately in the same range as the formaldehyde concentrations in the breathing zone in a steel foundry (2-4 ppm)^35^. The concentration of triethylamine in the breathing zones of core making operators was approximately higher than those (0.07-5.56 ppm) reported on 19 workers in 3 foundries^36^.

We found that FVC and FEV1 values were significantly lower in foundry workers than in control subjects. Our findings are consistent with those of Low and Mitchell^35^who found lower mean values of FVC among steel foundry workers. This finding also is in agreement with those of Johnson et al. ^37^who found significantly lower mean values of FEV1 among workers in an iron and steel foundry than control subjects did. In addition, our results are consistent with those of other findings and suggest that there was no difference in spirometric values of smokers and non-smokers in the case group^13^.This finding supports previous research, which stated the prime role of silica dust exposure in reducing ventilator capacity of the foundry workers^13^.

The results of this study revealed that combined exposure to silica dust and triethylamine had synergistic effects with some lung function parameters. This was accompanied by significant decreases in the values of FVC and FEV1. The phenomenon of simultaneous exposure of foundry workers to dust and fumes impaired their lung function parameters. In another study, combined occupational exposures to airborne dusts, gases, and fumes were associated with a reduction in peak expiratory flow rate (PEF)^38^. Although extensive research has been carried out to examine the synergistic effects of exposure to toxic agents^39, 40^, to the best of our knowledge, too little attention has been paid to determine the combined effects of respirable silica dust, formaldehyde, and triethylamine on lung function parameters in literature.

Prevalence of obstructive, restrictive, and mixed ventilatory impairments were significantly higher among our foundry workers than among control subjects. Although there were no statistically significant correlation between formaldehyde exposure and lung function parameters, the cumulative exposure of formaldehyde of our foundry workers was significantly correlated with FEF 25–75 after work shifts. This phenomenon signifies short and long-term effects of formaldehyde as irritant gas.

Results of combined exposure to silica dust and irritant gases on lung function parameters of foundry workers in this study, has raised serious concerns about their respiratory health. Therefore, more thorough examinations of their occupational exposures and health surveillance, including radiographic assessments of workers' lung conditions, according to Steenland and Ward (2014)^41^, more stringent of control measures, and health promotion programs are recommended for foundry workers.

## Conclusions

The mean concentrations of silica dust, formaldehyde, and triethylamine in the breathing- zone air of study foundry workers were higher than proposed occupational exposure limits. Some lung function parameters such as FVC and FEV1 values were significantly lower in foundry workers than in control subjects. The findings showed a statistically significant synergistic effect of silica dust and triethylamine on FVC values. Synergistic effects of exposure to formaldehyde and smoking habits on FEF 25–75 values were observed.

## Acknowledgements

This study was done as a partial fulfillment of Master of Science degree in Occupational Health Engineering. The authors also appreciate the workers and management of the foundry plant for their sincere supports and corporations.

## Conflict of interest statement

The authors have declared no conflict of interest.

## Funding

This project was financially supported by the Safety Promotion and Injury Prevention Research Center, Shahid Beheshti University of Medical Sciences, Tehran, Iran.

## Highlights

- The air concentrations of silica dust, formaldehyde, and triethylamine exceeded the respective threshold limit values.
- Lower values for some lung function parameters were observed in foundry workers compared to the control subjects.
- Synergistic effects of combined exposure to silica dust and triethylamine on FVC values were reported.
